# Tailoring the molecular design of twisted dihydrobenzodioxin phenanthroimidazole derivatives for non-doped blue organic light-emitting devices†

**DOI:** 10.1039/c8ra05004j

**Published:** 2018-08-14

**Authors:** Jayaraman Jayabharathi, Ramaiyan Ramya, Venugopal Thanikachalam, Pavadai Nethaji

**Affiliations:** Department of Chemistry, Annamalai University Annamalai Nagar Tamilnadu-608 002 India

## Abstract

Three fused polycyclic aryl fragments, namely, naphthyl, methoxynaphthyl, and pyrenyl have been used to construct blue-emissive phenanthroimidazole-functionalized target molecules, *i.e.*, 1-(2,3-dihydrobenzo[*b*][1,4]dioxin-6-yl)-2-(naphthalen-1-yl)-1*H*-phenanthro[9,10-*d*]imidazole (1), 1-(2,3-dihydrobenzo[*b*][1,4]dioxin-6-yl)-2-(1-methoxynaphthalen-4-yl)-1*H*-phenanthro[9,10-*d*]imidazole (2), and 1-(2,3-dihydrobenzo[*b*][1,4]dioxin-6-yl)-2-(pyren-10-yl)-1*H*-phenanthro[9,10-*d*]imidazole (3). The up-conversion of triplets to singlets *via* a triplet–triplet annihilation (TTA) process is dominant in these compounds due to 2*E*_T1_ > *E*_S1_. The pyrenyl dihydrobenzodioxin phenanthroimidazole (3)-based nondoped OLED exhibits blue emission (450 nm) with CIE (0.15, 0.14), a luminance of 53 890 cd m^−2^, power efficiency of 5.86 lm W^−1^, external quantum efficiency of 5.30%, and current efficiency of 6.90 cd A^−1^. The efficient device performance of pyrenyl dihydrobenzodioxin phenanthroimidazole is due to the TTA contribution to the electroluminescent process.

## Abstract 2

Efficient blue emitters, 1-(2,3-dihydrobenzodioxinyl)-2-naphthylphenanthroimidazole, 1-(2,3-dihydrobenzodioxinyl)-2-methoxynaphthylphenanthroimidazole and 1-(2,3-dihydrobenzodioxinyl)-2-pyrenylphenanthroimidazole have been reported.

## Graphical abstract

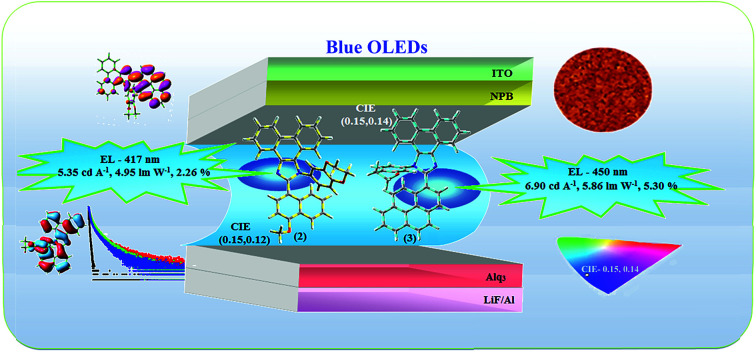

## 1. Introduction

Ever since the dawn of organic light-emitting diodes (OLEDs) Tang and coworkers reported there has been a sustained pursuit to develop high efficient, stable devices for applications capable of generating light of all visible colors.^1^ Although the research on OLEDs is far behind in commercial flat-panel displays with 100% internal quantum efficiencies (IQE) utilizing green and red electrophosphorescent devices,^2^ fabricating efficient blue OLEDs is of significant interest.^3^ However, the majority actual practical blue devices still rely on electroluminescence based on anthracene derivatives^4–6^ due to the longevity of triplet excitons, efficiencies reaching unity, generation of radiative decay (*k*_r_), which lead to quenching at high current density. White organic light-emitting diodes (WOLEDs) fabricated as a surface source in the lighting industry can provide light that is free from glare; however, WOLEDs rarely sustain efficiency at high brightness^7,8^ because of the poor performance of the blue component in WOLEDs. Therefore, exploiting blue OLEDs with excellent efficiencies at high brightness can increase the utility of OLEDs in flat-panel displays.^9^

According to spin statistics, IQE (internal quantum efficiency) of fluorescent OLEDs is limited (25%), but harvesting singlet and triplet excitons in the electroluminescence (EL) process is essential to produce highly efficient OLEDs. Additionally the TTA process (triplet–triplet annihilation) can generate a singlet exciton^10,11^ and triplet fusion (TF), resulting in enhanced efficiency.^12–15^ The TTA process can contribute 37.5% extra singlet excitons with radiative transition (*k*_r_) by modulating the molecular design.^15,16^ The high current density (*J*) is linearly related to the generation of an excess emissive portion *via* additional TF processes, which leads to high luminance with higher efficiency because of the TTA process.^17–19^ Therefore, various methods of using triplets can result in higher efficiency *via* TTA-based nondoped OLEDs,^20–24^ which are alternatives for the high-cost rare metal complex phosphorescent emitters.^25^ In blue OLEDs, where the TTA process is due to fused aromatics,^26–31^ the triplet energy is double the singlet energy, *i.e.*, 2*E*_T1_ > *E*_S1_.^32^

Phenanthroimidazole (PI) is a potential core unit for designing blue emitters because of its high quantum yield (PLQY) and balanced transport properties, which suppress the efficiency roll-off.^33–37^ PI-based derivatives with aryl substituents at the imidazole carbon cause extended π-conjugation and show intriguing electronic properties. The steric hindrance provided by the bulky substituent causes increased interchromophore packing, which in turn improves PLQY. However, planar emitting materials suffer from both inter- and intra-molecular interactions, resulting in bathochromic shifts. The quantum efficiency is low because of the closely packed and aggregated arrangement in the emitting layer.^38^ Blue emitters devoid of intermolecular interactions exhibit high PLQY. The increased carrier injection and transport properties of blue emitters are in high demand to develop blue OLEDs.^39,40^ Bulky side capping can prevent such interactions, thereby enhancing the formation of amorphous films. Therefore, it is desirable to incorporate the bulky side-capped dihydrobenzodioxin at the imidazole nitrogen to reduce the packing between the molecules, which limits emission quenching. This can enhance the stability of blue emission and also improve the quantum efficiency.^41,42^

To achieve maximum efficiencies while keeping the TTA process in view, fused polycyclic aryl dihydrobenzodioxin phenanthroimidazole derivatives, namely, 1-(2,3-dihydrobenzo[*b*][1,4]dioxin-6-yl)-2-(naphthalen-1-yl)-1*H*-phenanthro[9,10-*d*]imidazole (1), 1-(2,3-dihydrobenzo[*b*][1,4]dioxin-6-yl)-2-(1-methoxynaphthalen-4-yl)-1*H*-phenanthro[9,10-*d*]imidazole (2), and 1-(2,3-dihydrobenzo[*b*][1,4]dioxin-6-yl)-2-(pyren-10-yl)-1*H*-phenanthro[9,10-*d*]imidazole (3) were synthesized and used as emissive layers in the device configuration of ITO/NPB (*N*,*N*′-di-(1-naphthyl)-*N*,*N*′-diphenyl-(1,1′-biphenyl)-4,4′-diamine)benzidine)/polycyclic aryl dihydrobenzodioxin phenanthroimidazole derivatives (1–3)/Alq_3_ (tris(8-hydroxyquinoline)aluminum)/LiF/Al.^24^ A device with 1-(2,3-dihydrobenzo[*b*][1,4]dioxin-6-yl)-2-(pyren-10-yl)-1*H*-phenanthro[9,10-*d*]imidazole (3) as the emissive material showed a maximum current efficiency (*η*_c_) of 6.90 cd A^−1^, power efficiency (*η*_p_) of 5.86 lm W^−1^, external quantum efficiency (*η*_ex_) of 5.30%, and luminance of 53 890 cd m^−2^. Since a pyrenyl substituent at the imidazole carbon (C25) of phenanthroimidazole can extend π-conjugation, exhibit efficient electronic properties, and provide effective steric hindrance in the interchromophore packing and TTA contribution, the results showed enhanced quantum efficiency.

## 2. Experimental section

### 2.1. Fused polycyclic aryl dihydrobenzodioxin phenanthroimidazoles (1–3)

Phenanthrene-9,10-dione (1 mmol), fused polycyclic aryl aldehyde (1 mmol), 1,4-benzodioxane-6-amine (1 mmol), and ammonium acetate (1 mmol), all in acetic acid (25 ml), were refluxed (120 °C; 24 h). The phenanthroimidazole solution was chilled, and the crude product was separated by column chromatography (Scheme S1†). The purified structures of the fused polycyclic aryl dihydrobenzodioxin phenanthroimidazoles 1–3 were confirmed with elemental analysis, ^1^H (Fig. S1–S3†)/^13^C NMR (Fig. S4–S6†) spectra, and mass spectrometry (Fig. S7–S9†).

#### 2.1.1. 1-(2,3-Dihydrobenzo[*b*][1,4]dioxin-6-yl)-2-(naphthalen-1-yl)-1*H*-phenanthro[9,10-*d*]imidazole (1)

Yield 60%. Anal. calcd: C_33_H_22_N_2_O_2_: C, 82.83; H, 4.63; N, 5.85. Found: C, 82.38; H, 4.59; N, 5.58. 400 MHz ^1^H NMR (CDCl_3_): *δ* 4.17–4.26 (m, 4H), 6.83 (d, *J* = 8.0 Hz, 2H), 7.12 (d, *J* = 7.6 Hz, 1H), 7.35 (s, 2H), 7.42–7.48 (m, 2H), 7.65–7.74 (m, 2H), 7.86 (d, *J* = 8.4 Hz, 2H), 7.96 (d, *J* = 8.0 Hz, 2H), 8.16 (d, 1H), 8.72–8.75 (m, 3H), 9.68–9.66 (d, *J* = 8.4 Hz, 1H). 100 MHz ^13^C NMR (CDCl_3_): *δ* 63.32, 63.35, 105.63, 114.67, 115.96, 122.42, 123.74, 125.32, 127.58, 128.04, 129.56, 130.98, 131.74, 132.55, 133.38, 134.26, 136.28, 146.45, 147.65, 148.36, 149.34. MS: *m*/*z*. 478.17 [M^+^]. Calcd: 478.21.

#### 2.1.2. 1-(2,3-Dihydrobenzo[*b*][1,4]dioxin-6-yl)-2-(1-methoxynaphthalen-4-yl)-1phenanthro[9,10-*d*]imidazole (2)

Yield 52%. Anal. calcd: C_34_H_24_N_2_O_3_: C, 80.30; H, 4.76; N, 5.51. Found: C, 80.22; H, 4.65; N, 5.49. 400 MHz ^1^H NMR (CDCl_3_): *δ* 3.95 (s, 3H), 4.05–4.25 (m, 4H), 6.78 (d, *J* = 8.4 Hz, 2H), 6.88 (d, *J* = 8.0 Hz, 2H), 7.28 (d, 1H), 7.52–7.62 (m, 4H), 7.82 (d, 1H), 8.26 (d, *J* = 8.4 Hz, 1H), 8.56 (d, *J* = 8.4 Hz, 2H), 8.69 (d, *J* = 8.0 Hz, 2H), 9.24 (s, 2H). 100 MHz ^13^C NMR (CDCl_3_): *δ* 55.21, 64.12, 64.55, 101.63, 104.71, 113.56, 115.42, 122.14, 123.32, 125.38, 126.94, 127.56, 128.38, 129.64, 130.81, 131.38, 132.26, 133.28, 136.25, 145.35, 146.36, 149.34. MS: *m*/*z*. 508.18 [M^+^]. Calcd: 509.18.

#### 2.1.3. 1-(2,3-Dihydrobenzo[*b*][1,4]dioxin-6-yl)-2-(pyren-10-yl)-1*H*-phenanthro[9,10-*d*]imidazole (3)

Yield 55%. Anal. calcd: C_39_H_24_N_2_O_2_: C, 84.76; H, 4.38; N, 5.07. Found: C, 84.55; H, 4.15; N, 5.01. 400 MHz ^1^H NMR (CDCl_3_): *δ* 4.24–4.33 (m, 4H), 6.97 (s, 2H), 7.41–7.38 (m, 4H), 7.68 (s, 2H), 7.76–7.81 (m, 6H), 7.92 (s, 1H), 8.01–8.07 (m, 3H), 8.98 (s, 2H). 100 MHz ^13^C NMR (CDCl_3_): *δ* 64.32, 64.25, 101.63, 114.37, 115.66, 121.92, 122.04, 124.92, 125.78, 126.64, 127.56, 129.68, 131.54, 133.45, 134.18, 146.96, 147.38, 149.25. MS: *m*/*z*. 552.18 [M+]. Calcd: 553.19.

### 2.2. Measurements and general methods

The reagents and solvents used for the synthesis were purchased from commercial sources. The ^1^H and ^13^C NMR results were recorded with a Bruker 400 MHz spectrometer, and mass spectrometry results were recorded on Agilent (LCMS VL SD). The UV-vis absorptions of 1–3 in solution and film were measured on a Perkin-Elmer Lambda 35 and Lambda 35 spectrophotometer with an integrated sphere (RSA-PE-20) instrument, respectively. Photoluminescence (PL) studies were carried out with a Perkin-Elmer LS55 fluorescence spectrometer. Thermogravimetric analysis (TGA) and differential scanning calorimetry (DSC) were carried out using the Perkin-Elmer thermal analysis system and NETZSCH-DSC-204, respectively; the heating rate for TGA and DSC was 10 °C min^−1^, and the N_2_ flow rate was 100 mL min^−1^. The fluorescence lifetimes of the emissive materials 1–3 (toluene solution, ×10^−5^ mol L^−1^) were calculated from the time-resolved fluorescence decays obtained by the time-correlated single-photon counting (TCSPC) method on the Horiba Fluorocube-01-NL lifetime system with a nano LED as the excitation source and TBX-PS as the detector. The fluorescence decay was analysed by the reconvolution method using the DAS6 software provided by Horiba Fluorocube-01-NL instruments. The goodness of fit was determined using the reduced *χ*^2^ values. PLQY (quantum yield) was determined with quinine sulphate (0.54) as the reference 
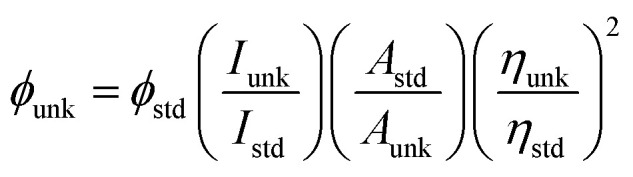
, where *ϕ*_unk_ and *ϕ*_std_ are the QYs of unknown and standard materials, respectively; *I*_unk_ and *I*_std_ are the emission intensities of unknown and standard materials, respectively; *A*_unk_ and *A*_std_ are the absorbances of unknown and standard materials, respectively; and *η*_unk_ and *η*_std_ are the refractive indexes of unknown and standard materials, respectively]. Cyclic voltammetry was scanned with a potentiostat CHI 630A electrochemical analyzer (platinum electrode and platinum wire as the working electrode and counter electrode, respectively, Ag/Ag^+^ as the reference electrode, scan rate of 100 mV s^−1^). Ferrocene was used as the internal standard with the highest occupied molecular orbital energy of −4.80 eV, and 0.1 M tetrabutylammoniumperchlorate in CH_2_Cl_2_ was used as the supporting electrolyte. The HOMO energies were calculated by measuring the oxidation potentials [*E*_HOMO_ = −(*E*_ox_ + 4.8 eV)], and the LUMO energies were estimated by subtracting from the HOMO energies with the optical band gap [*E*_LUMO=_E_HOMO_ − 1239/*λ*_onset_]. For theoretical calculations, the geometrical properties were optimized at the B3LYP/6-31 g (d, p) level using the Gaussian 09 program.^43^

### 2.3. Device fabrication and measurement

Devices with a configuration of ITO/NPB (60 nm)/fused polycyclic aryl dihydrobenzodioxin phenanthroimidazoles 1–3 (20 nm)/Alq_3_ (30 nm)/LiF (1 nm)/Al (100 nm), hole-only devices [ITO/HATCN(10 nm)/NPB (20 nm)/1–3 (60 nm)/NPB (20 nm)/Al(100 nm)], and electron-only devices [ITO/TPBi (10 nm)/1–3 (60 nm)/TPBi (10 nm)/LiF (1 nm)/Al (100 nm)] were fabricated on pre-cleaned ITO-coated glass substrates with a sheet resistance of 20 Ω sq^−1^. Current density–voltage characteristics were recorded with a Keithley 2400 power source. The EL spectra and CIE coordinates were measured with a spectrometer (USB-650-VIS-NIR, Ocean Optics, Inc., USA).

## 3. Results and discussion

Fused polycyclic aryl phenanthroimidazoles, namely, 1-(2,3-dihydrobenzo[*b*][1,4]dioxin-6-yl)-2-(naphthalen-1-yl)-1*H*-phenanthro[9,10-*d*]imidazole (1), 1-(2,3-dihydrobenzo[*b*][1,4]dioxin-6-yl)-2-(1-methoxynaphthalen-4-yl)-1*H*-phenanthro[9,10-*d*]imidazole (2), and 1-(2,3-dihydrobenzo[*b*][1,4]dioxin-6-yl)-2-(pyren-10-yl)-1*H*-phenanthro[9,10-*d*]imidazole (3) with side-capped dihydrobenzodioxin were synthesized by the Debus–Radziszewski reaction with appreciable yields. The synthetic route for the emissive materials is displayed in Scheme S1.†

### 3.1. Potential energy surface (PES) scan studies and thermal properties

**Fig. 1:**
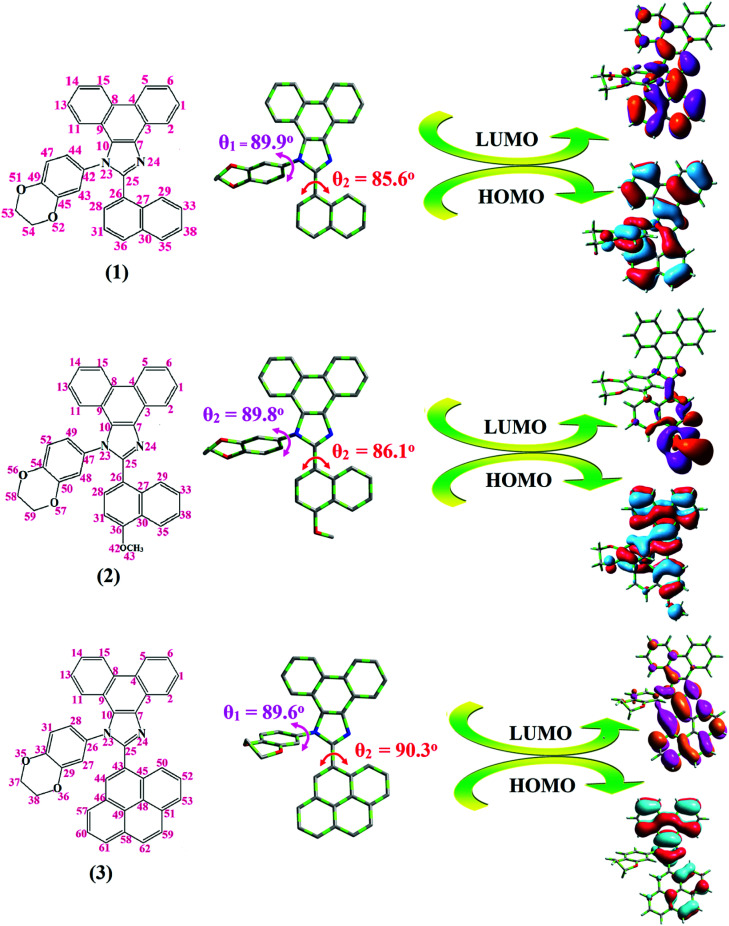
Molecular structure, optimised geometry, and frontier molecular orbitals of fused polycyclic aryl dihydrobenzodioxin phenanthroimidazoles.

**Fig. 2:**
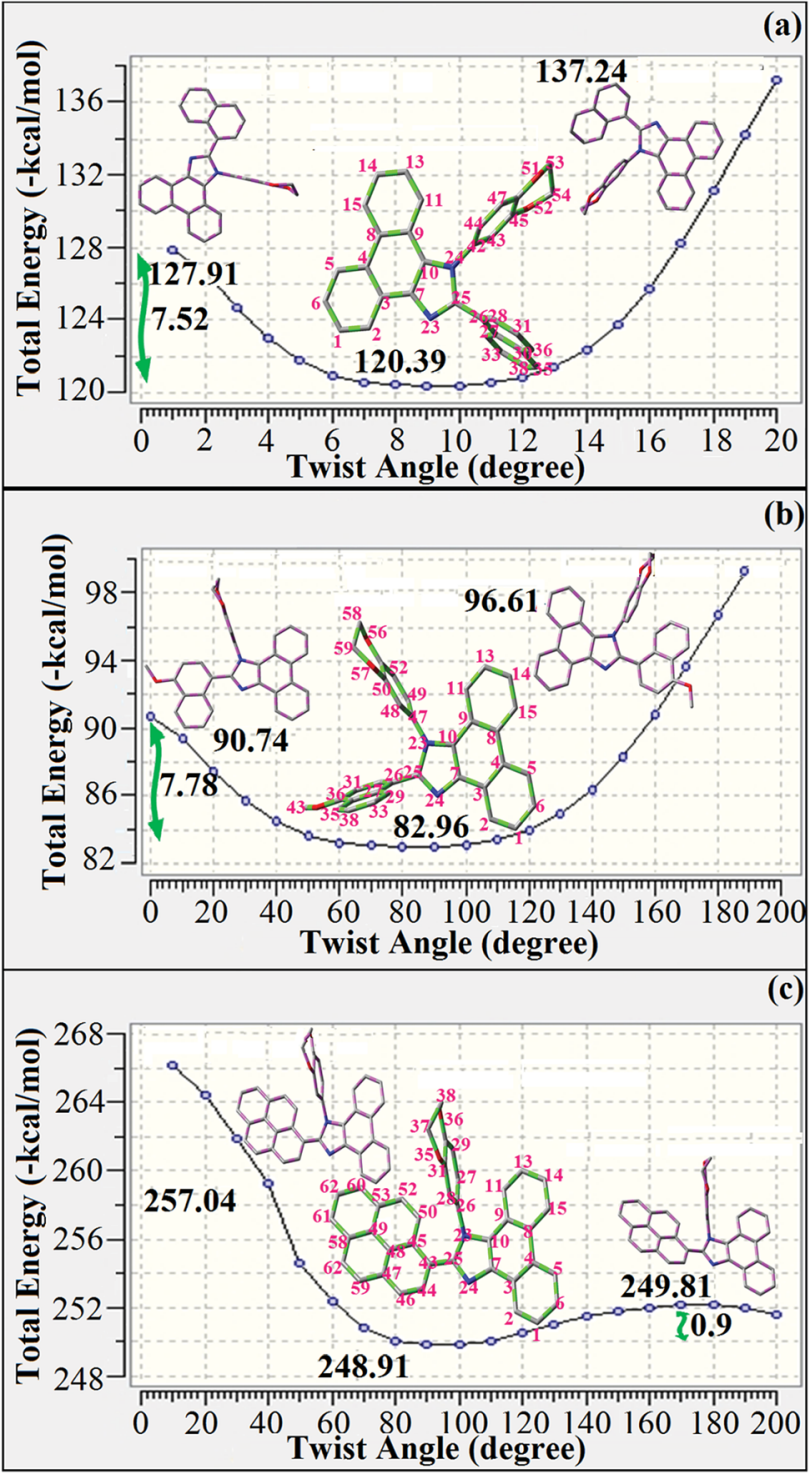
Potential energy surface scan diagram of fused polycyclic aryl dihydrobenzodioxin phenanthroimidazoles [a (1), b (2), and c (3)].

**Fig. 3:**
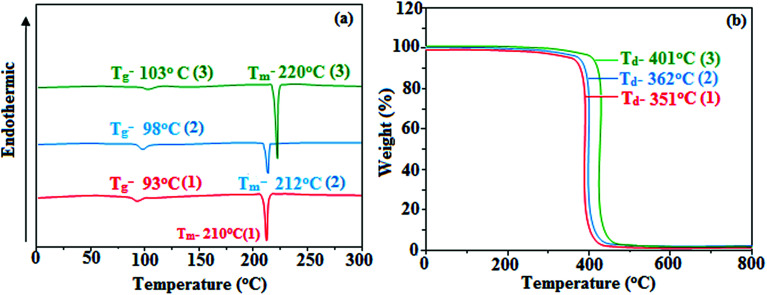
(a) DSC and (b) TGA graphs of fused polycyclic aryl dihydrobenzodioxin phenanthroimidazoles.

Correlations of the emissive material configurations with their physical properties were analyzed theoretically, and the electronic structures of compounds 1–3 were optimized using DFT at the B3LYP/6-31G (d) level. The electron-cloud orientation of the frontier molecular orbitals is schematically displayed in Fig. 1. Potential energy surface scans for N24–C25–C26–C28 (1)/N24–C25–C26–C28 (2)/N24–C25–C43–C44 (3) were performed; during the calculations, all the geometrical parameters were simultaneously relaxed, whereas the torsional angles were varied in steps of 0°, 20°, 40°, 60°…360°. The potential energy surface diagram revealed that the minimum energy conformation corresponded to the one in which the dihydrobenzodioxin ring was attached orthogonally to the imidazole nitrogen atom (N23) (Fig. 2). The twisted fused polycyclic aryl dihydrobenzodioxin phenanthroimidazoles could effectively suppress the molecular aggregation; the almost orthogonal dihedral angles (∼89.0°) between dihydrobenzodioxin and the phenanthrimidazole core effectively minimized the intermolecular packing, and they could be used as hole-trapping sites, whereas the peripheral phenanthrimidazole core blocked the electron-trapping sites. Thus, effective carrier injection as well as transport ability could be expected from these reported emitters; the relative charge carrier transport abilities of the title materials 1–3 were investigated in hole-only devices as well as in electron-only devices. The introduction of a dihydrobenzodioxin group on the phenanthroimidazole unit could enhance the molecular distortion degree, thereby suppressing the formation of aggregation or π–π stacking in the solid state and forming an amorphous film (smooth and pinhole-free) during device fabrication.^44^ The side capping of dihydrobenzodioxin at N23 was twisted with respect to the phenanthroimidazole frame with dihedral angles of 89.9° (1)/89.8° (2)/89.6° (3). The fused aryl group attached to the imidazole carbon atom (C25) was tilted at angles of 85.6° (1)/86.1° (2)/90.3° (3), and these dihedral angles confirmed the non-coplanar twisting configurations of 1–3.^45–47^ The non-planar conformation may effectively suppress the red shift and maintain quantum efficiency in the film *via* restraining the intermolecular interaction.^19^ The incorporation of the bulky fragments of naphthyl, methoxynaphthyl, and pyrenyl and side-capped dihydrobenzodioxin into phenanthroimidazoles enlarged their sizes and improved their thermal stabilities. To achieve high quantity of heat liberation with high current density (*J*) in EL devices, blue-emissive phenanthroimidazoles with maximum thermal stability should be used (Table 1). Glass transition temperatures (*T*_g_) of 93 °C, 98 °C, and 103 °C were observed for the dihydrobenzodioxin phenanthroimidazoles 1–3, respectively, and their melting temperatures measured by DSC were 210 °C (1), 212 °C (2), and 220 °C (3) (Fig. 3a). The capping of the bulky dihydrobenzodioxin moiety at the imidazole nitrogen increased the glass transition temperature when compared with phenyl substitution at the imidazole nitrogen.^48^ This was most likely due to the highly rigid and twisted structure, which might suppress the intermolecular interaction between these molecules in film. The decomposition temperatures (*T*_d_) of compounds 1–3 were measured as 351 °C, 362 °C, and 401 °C, respectively (Fig. 3b). High *T*_d_ of dihydrobenzodioxin phenanthroimidazoles indicated the high resistance of the fused aromatic rings on thermolysis; high *T*_m_ and *T*_d_ could enhance the lifetimes of devices,^49–51^ and the formation of a stable film can be expected upon device fabrication. The dihydrobenzodioxin phenanthroimidazole derivatives exhibited smooth surface morphology with a roughness of 0.28 nm (Fig. 7d), which remained unchanged even after annealing (90 °C, 6 h); this also proves the suitability of these blue-emissive materials for device fabrication.

Photophysical and thermal properties and device efficiencies

**Table:** 

Parameters	1	2	3
Photophysical and thermal properties			
*T*_g_/*T*_m_/*T*_d_ (°C)	93/210/351	98/212/362	103/220/401
*λ*_ab_(nm) (asol/bfilm)	244, 295, 362/368	240, 303, 364/370	252, 283, 352/358
c *Φ*_PL_ (asol/bfilm)	60/62	63/68	68/49
*τ* (ns) (sol/film)	2.24/1.80	2.31/1.86	2.64/1.92
*λ*_emi_ (nm) (asol/bfilm)	378, 400/404	348, 416/418	351, 440/452
d *E*_T_ (eV)	3.10	2.98	2.82
*k*_r_ × 10^8^ (s^−1^)	2.6	2.7	2.5
k_nr_ × 10^8^ (s^−1^)	1.8	1.6	1.2
eHOMO/LUMO (eV)	−5.36/−2.20	−5.30/−2.26	−5.29/−2.49
*E*_g_ (eV)	3.16	3.04	2.80

a Toluene solution (×10^−5^ mol L^−1^).

b Neat film by coating.

c Absolute PLQY measured with integrating sphere.

d Triplet energy calculated from the first vibrational peak of phosphorescent spectrum.

e *E*_HOMO_ = −(*E*_ox_ + 4.8 eV); *E*_LUMO_ = *E*_HOMO_ − 1239/*λ*_onset_.

**Table:** 

Device efficiency	I	II	III
*V*_on_ (V)	3.9	3.8	3.1
EL (nm)	404	417	450
*L* (cd m^−2^)	36 823	38 032	53 890
*η*_ex_ (%)	2.13	2.26	5.30
*η*_c_ (cd A^−1^)	5.10	5.35	6.90
*η*_p_ (lm W^−1^)	4.82	4.95	5.86
CIE (*x*,*y*)	0.16,0.13	0.15,0.12	0.15,0.14

### 3.2. Photophysical properties

**Fig. 4:**
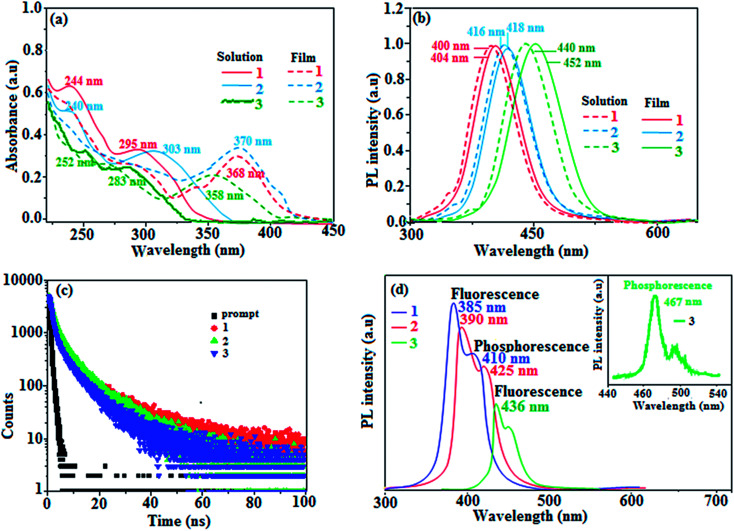
(a) Absorption, (b) emission, (c) lifetime, and d) delayed PL spectra of fused polycyclic aryl dihydrobenzodioxin phenanthroimidazoles.

Absorption (*λ*_abs_) and emission (*λ*_emi_) spectra were measured in dichloromethane and thin films on quartz substrates to explore the ground and excited state properties of the dihydrobenzodioxin phenanthroimidazoles 1–3 (Fig. 4). Since the core fragment was the same, compounds 1–3 exhibited identical absorptions at 244, 295, 362 (1), 240, 303, 364 (2), and 252, 283, 352 nm (3). The absorption from 303 to 364 nm was due to the delocalized π–π* transition of phenanthroimidazole,^52^ and absorption from 244 to 303 nm may due to the combination of π–π* transitions of the aromatic segments.^53,54^ Compared to the observations for naphthyl and methoxynaphthyl dihydrobenzodioxin phenanthrimidazoles (1 and 2), the vibrational absorption band (283 nm) of pyrenyl dihydrobenzodioxin phenanthrimidazole (3) was due to π–π* transition of the pyrenyl fragment. The stronger molar absorption coefficient (2.7 × 10^4^ L mol^−1^ cm^−1^) revealed that more conjugation was favorable for a higher quantum yield (*Φ*). The quantum yield of these compounds in dilute THF (10^−5^ mol L^−1^) was measured using quinine sulphate in 1 mol L^−1^ sulphuric acid as a standard. The fluorescent quantum yields of dihydrobenzodioxin phenanthrimidazoles were measured as 60, 63 and 68% for 1–3, respectively; the higher quantum yield was due to the presence of naphthyl, methoxynaphthyl, and pyrenyl at the imidazole carbon, which could impart enhanced conjugation, resulting in radiative transition.^55^ The fluorescence lifetimes [2.24 ns (1), 2.31 ns (2), and 2.64 ns (3): Fig. 4c] and quantum yields in the film were measured [62% (1), 68% (2), and 49% (3)] to predict the suitability for device fabrication (Table 1). The decay curve revealed that no delayed lifetime component was detected with a prompt lifetime of less than 2 ns (Fig. 4), and a short lifetime was speculated from the fast non-radiative decay rate.^56^

**Fig. 5:**
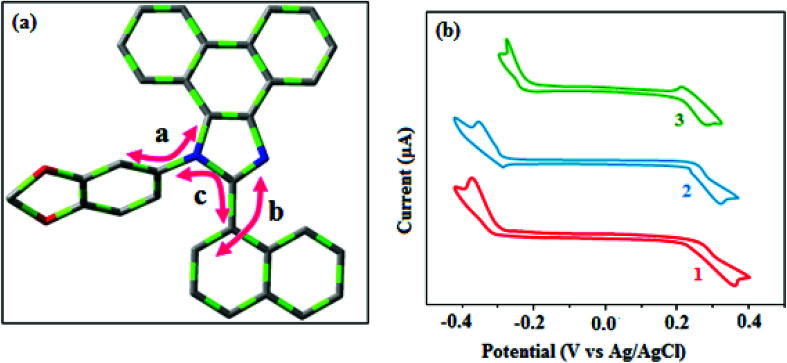
(a) Schematic representation of the dihedral angles and (b) cyclic voltammogram of fused polycyclic aryl dihydrobenzodioxin phenanthroimidazoles.

Generally, photoluminescence quantum yield depends on the radiative transition rate (*k*_r_), and the total irradiative transition rate (Σ*k*_ir_) can be described as *Φ* = *k*_r_/(*k*_nr_ + Σ*k*_ir_).^31,57^ The low value of PLQY of 49% for 3 in film may be due to concentration quenching resulting from intermolecular aggregation, causing energy loss during the excited process. This may increase Σ*k*_ir_ and lead to a smaller quantum yield, which is in agreement with the results reported for pyrenyl derivatives.^58^ The triplet excited state properties of twisted fused polycyclic aryl dihydrobenzodioxin phenanthroimidazoles (1–3) can be studied by steady state phosphorescent emission spectral analysis. The steady state phosphorescent emissions of naphthyl and methoxynaphthyl dihydrobenzodioxin phenanthroimidazoles (1 and 2) are recorded at 77 K; the results show phosphorescent peaks at 410 and 425 nm, respectively, combined with fluorescence [385 nm (1) and 390 nm (2)], and their corresponding triplet state energies (*E*_T1_) are 3.01 eV (1), 2.92 eV (2), and 2.10 eV (3). However, by directly exciting pyrenyl dihydrobenzodioxin phenanthroimidazoles (3) with a proper laser flash, the triplet emission is not accessible at the same experimental condition (Fig. 4d); thus, time-resolved PL is carried out to measure the delayed emission from phosphorescence, and the delayed emission is found to be centered at 467 nm (2.65 eV).^51^ The calculated triplet energy reveals that the lowest triplet excited states are localized on the fused aryl fragments, namely, naphthyl, methoxynaphthyl, and pyrenyl groups for compounds 1, 2, and 3, respectively.^59,60^ Their singlet excited state energies (*E*_S1_) can also be extrapolated from steady state fluorescence emission at 77 K [385 nm (3.22 eV) (1), 390 nm (3.12 eV) (2), and 436 nm (2.84 eV) (3)]. According to the absorption onset, the optical gaps of these compounds are calculated to be 3.16 eV, 3.04 eV, and 2.80 eV. Since 2*E*_T1_ > *E*_S1_, the up-conversion of triplets → singlets *via* the TTA process is predominant in these compounds. The high triplet energy and balanced carrier-transport ability of fused polycyclic dihydrobenzodioxin phenanthrimidazoles are beneficial for electron–hole recombination within the emissive layer.^61^ The aggregation of fused polycyclic dihydrobenzodioxin phenanthrimidazoles shows a marked variation of absorption (*λ*_abs_) and emission (*λ*_emi_) in the solution/film (Fig. 4). The smaller dihedral angle between the fused aryl ring incorporated with the imidazole carbon and the dihydrobenzodioxin moiety attached to azomethane nitrogen induces π → π* stacking interaction in the film and causes a red shift (Table 1: Fig. 5a). The radiative (*k*_r_) and non-radiative (*k*_nr_) rate constants of the dihydrobenzodioxin phenanthroimidazoles [*k*_r_ = *ϕ*/τ; *k*_nr_ = 1/*τ* − (*ϕ*/*τ*): Table 1] reveal that *k*_r_ > *k*_nr_. The small HOMO^54^ energies of −5.36 eV (1), −5.30 eV (2), and −5.29 eV (3) estimated from their respective oxidation onset potentials [0.56 V (1), 0.50 V (2), and 0.49 V (3)] support the hole injection ability of dihydrobenzodioxin phenanthroimidazoles (Fig. 5b: Table 1). The LUMO energies of −2.20 eV (1), −2.26 eV (2), and −2.49 eV (3) are close to that of 1,3,5-tris(*N*-phenylimidazol-2-yl)benzene (−2.40 eV), supporting the electron injection abilities of the dihydrobenzodioxin phenanthroimidazoles. The frontier molecular orbital analysis confirms the carrier injection abilities of the dihydrobenzodioxin phenanthroimidazoles, indicating that they can be used as potential emitters in OLEDs.^62^ The molecular orbital distribution shows that the HOMOs are delocalized dominantly over the entire molecule, whereas the LUMOs are concentrated on the fused aryl group, indicating the charge separation between HOMO and LUMO (Fig. 1). Such orbital separation is more predominant in 1-(2,3-dihydrobenzo[*b*][1,4]dioxin-6-yl)-2-(pyren-10-yl)-1*H*-phenanthro[9,10-*d*]imidazole (3), which is beneficial for achieving effective hole and electron transport in optoelectronic devices.^46,63^

### 3.3. Electroluminescence properties

**Fig. 6:**
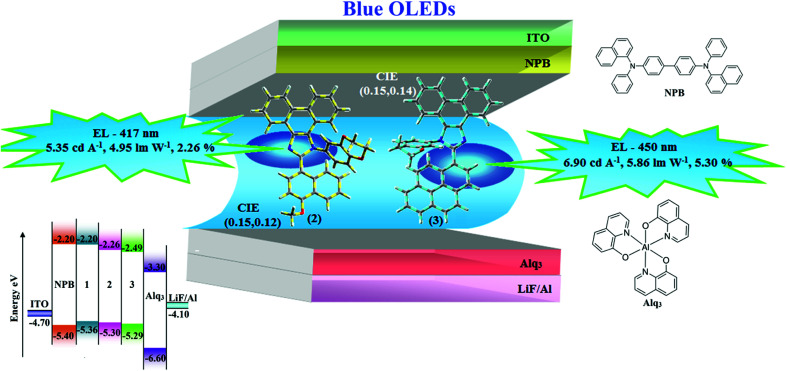
Energy level and molecular structures of materials used in devices.

**Fig. 7:**
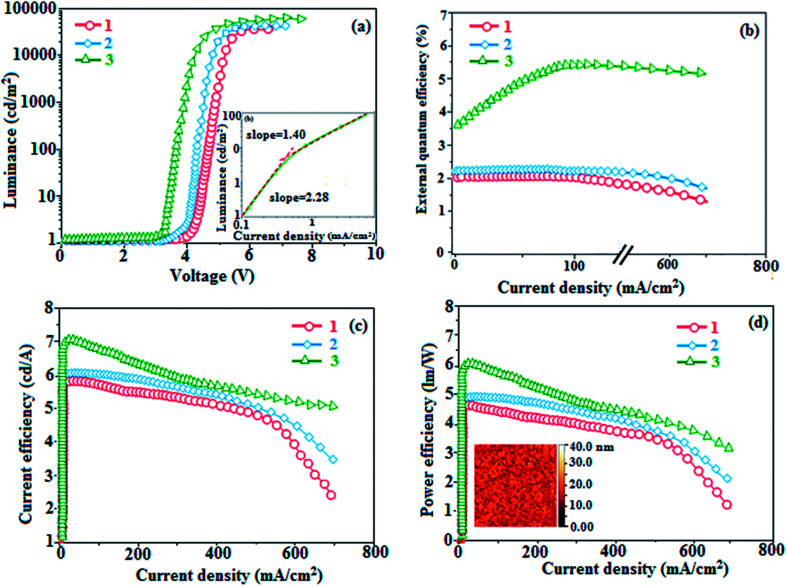
Device performances: (a) luminance (*L*) *versus* voltage [inset: roll-off efficiency, device III]; (b) external quantum efficiency (*η*_ex_) *versus* current density (*J*); (c) current efficiency (*η*_c_) *versus* current density (*J*), and (d) power efficiency (*η*_p_) *versus* current density (*J*) [inset: AFM image].

**Fig. 8:**
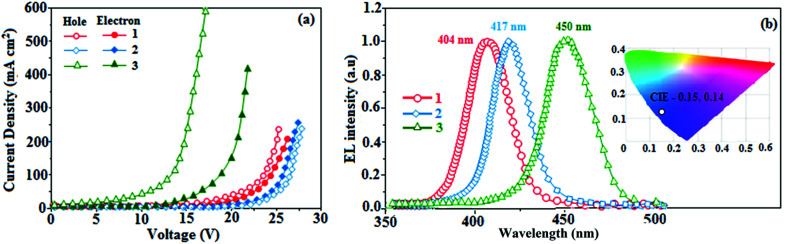
(a) Hole-only (IV) and electron-only (V) devices of fused polycyclic aryl dihydrobenzodioxin phenanthrimidazoles and (b) electroluminescent spectra of fused polycyclic aryl dihydrobenzodioxin phenanthroimidazoles [inset: CIE coordinates of device III].

**Fig. 9:**
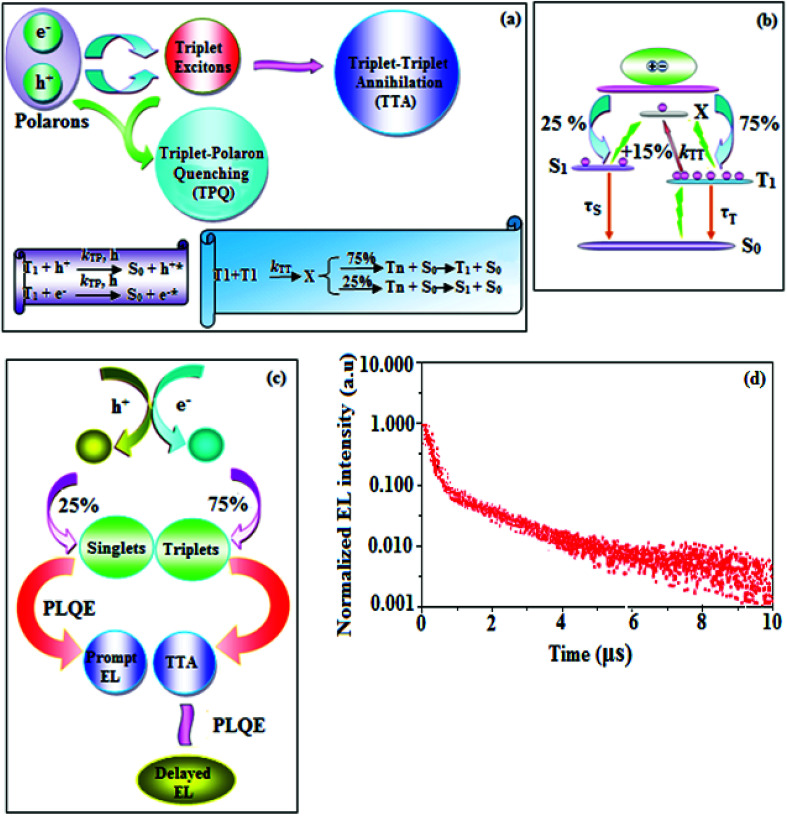
Schematic representation of (a) triplet–triplet annihilation (TTA)/triplet-polaron quenching (TPQ) processes, (b) spin-statistics in electrically driven OLEDs, (c) delayed emission and (d) transient-EL curve for device III.

To explore the fused polycyclic aryl dihydrobenzodioxin phenanthroimidazoles for blue-emissive material devices with a configuration of ITO/NPB (60 nm), fused polycyclic aryl dihydrobenzodioxin phenanthroimidazoles 1–3 (20 nm)/Alq_3_ (30 nm)/LiF (1 nm)/Al (100 nm) were fabricated (Fig. 6), where NPB was used as the hole injection and transportation layers, and Alq_3_ and LiF were used as the electron transporting and electron injection layers, respectively. The fabricated devices (I–III) showed emission in the range of 404–450 nm and exhibited high brightness (Fig. 8); their observed EL spectra were consistent with the PL film spectra, which indicated that the emission originates only from the emissive layer without emission from the interface exciplex. The device III with the emissive layer of 1-(2,3-dihydrobenzo[*b*][1,4]dioxin-6-yl)-2-(pyren-10-yl)-1*H*-phenanthro[9,10-*d*]imidazole exhibited emission at 450 nm with a current efficiency (*η*_c_) of 6.90 cd A^−1^, power efficiency (*η*_p_) of 5.86 lm W^−1^, external quantum efficiency (η_ex_) of 5.30%, and luminance of 53 890 cd m^−2^ (Fig. 7). Devices I and II exhibited stable external quantum efficiencies with negligible roll-off efficiencies due to balanced carrier mobilities,^34,64^ whereas device III showed a roll-off efficiency curve at low current density (<9 mAcm^−2^). DFT calculations revealed that the formed triplet excitons were probably proportional to the current density (*J*), assuming that the recombination efficiency was 100% in the emissive region. The concentration of triplet exciton is related to the TTA probability as a quadratic function with respect to *J* at low current density, leading to TTA emission, which is linearly related to the square of the current density (*J*).^49^ Moreover, the quadratic TTA emission on the current density becomes linear as the TTA probability tends to saturate at high current density (TTA emission probably saturated). Though it is impossible to split the TTA emission from EL luminance, the luminance curve of device III in Fig. 7 revealed non-monoexponential relation with the quadratic TTA-emission. Both the theoretical and experimental results confirmed that the quadratic nature of the TTA emission at low current density changed to a linear nature at high current density.^65^ However, no TTA contribution was predicted in devices I and II, probably due to their high *E*_T1_ values to fuse the double energy excited state. The side group dihydrobenzodioxin was twisted with respect to the phenanthroimidazole frame with orthogonal dihedral angles of 89.9° (1) and 89.8° (2), which effectively minimized the intermolecular packing and thus, these could be used as hole-trapping sites, whereas the peripheral phenanthrimidazole core blocked the electron-trapping sites. Thus, effective carrier recombination in the emissive layer resulted in higher EL performances. The triplet population depletion was analysed in EL processes by the fabrication of hole-only and electron-only devices with configurations of ITO/HATCN(10 nm)/NPB (20 nm)/1–3 (60 nm)/NPB (20 nm)/Al(100 nm) (hole-only device IV): (c) ITO/TPBi(10 nm)/1–3 (60 nm)/TPBi(10 nm)/LiF(1 nm)/Al (100 nm) (electron-only device V). For the hole-only device, NPB adjacent to Al was deposited to block electrons, whereas in the electron-only device, TPBi close to the anode was deposited to avoid hole injection.^66^ It was found that the carrier mobility of compound 3 was higher than those of compounds 1 and 2 because of the pyrenyl fragment in compound 3 (Fig. 8). The stronger hole-transporting property revealed that the exciton deactivation was mainly quenched by cation radicals accumulated in the emissive region, resulting in the TPQ process (triplet-polaron quenching).^67^ The decrease in carrier concentration may be expected to lower the operational voltage and also to enhance the TTA process, thus improving the EL efficiency (Fig. 9a). TTA is a diffusion-limited process with the collision of two triplets, leading to an intermediate state X with twice the energy as that of the T_1_ state. The intermediate state X comprises one singlet (1/4), three triplets (3/4), and five quintet states; the quintet states are generally expected to be higher in energy than the two triplets and thus can be neglected. The intermediate state then relaxes to either an excited triplet state (T_*n*_), which rapidly relaxes to a T_1_ triplet, or to a singlet state (S_1_), and the possible equation is as follows:
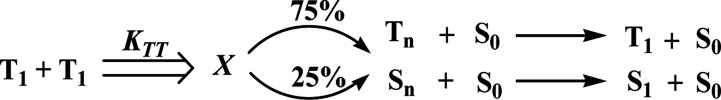

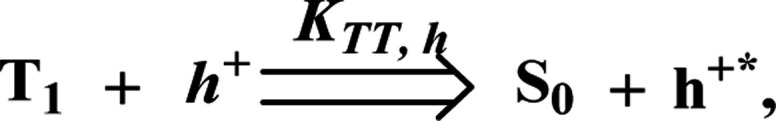

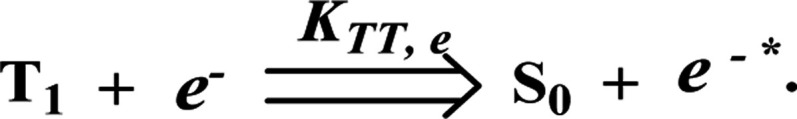
Here, *k*_TT_ is the rate constant of the TTA process. The total fraction of triplet excitons converted to singlet excitons through multiple TTA processes is 15%,^68^ and TTA in fluorescent materials may increase the device efficiency (Fig. 9b). The singlet exciton ratio is calculated to determine the TTA contribution using the equation EQE = *γη*_r_*ϕ*_PL_*η*_out_, where *γ* is the carrier recombination efficiency [100%], *η*_r_ is the singlet exciton ratio, *η*_out_ is the light out-coupling efficiency (20–25% for blue OLEDs),^69,70^ and *ϕ*_PL_ is the photoluminescence quantum efficiency [49% for the pyreneyl phenanthrimidazole film]. The value of η_r_ for device III is calculated to be 36-45%, which indicates that *γ* is less than 100% due to unbalanced carrier transportation.^49^ To explain the higher external quantum efficiency of device III than the classic upper limit (5%), the transient electroluminescence of device III is measured.

Fluorescence emission takes place in the TTA process *via* the excited triplet state in a microsecond timescale, which is longer than that for normal emission, which occurs in the nanosecond timescale. Time-resolved electroluminescent measurement was recorded for device III based on 1-(2,3-dihydrobenzo[*b*][1,4]dioxin-6-yl)-2-(pyren-10-yl)-1*H*-phenanthro[9,10-*d*]imidazole (3) to support the TTA process (Fig. 9c), and device III showed delayed fluorescence with a decay time of 3.9 μs (Fig. 9d). The delayed fluorescence contribution to the total light emission was evaluated by extrapolating the transient decay curves, and it was found that device III exhibited a contribution of ∼10%. This result indicated that the triplet exciton density of the emissive layer host was appreciable to cause TTA in device III because the triplet excitons generated in the emissive layer diffused to the hole-transport layer owing to the lower *E*_T_ level for the hole-transport material NPB (*E*_T_ = 2.47 eV) than that for pyrenyl dihydrobenzodioxin phenanthroimidazole (3) (*E*_T_ = 2.65 eV). The characteristic of device III suggested a correlation between the probability of TTA and the external quantum efficiency. This correlation showed that the high efficiency of device III resulted from the TTA-induced delayed fluorescence. On the other hand, TTA occurred only at a rate of ∼10% in device III, giving 5.30% external quantum efficiency. The transient life time and external quantum efficiency results were employed in the formula reported by Di *et al.*^71^ to calculate the percentage of TTA: *η*_TTA_ = 2*f*_delayed_ EQE/*f*_excitons_*f*_triplets_*f*_outcoupling_ PLQE, where *f*_delayed_ is the % of delayed EL, *f*_outcoupling_ is the outcoupling efficiency of the emitter (assumed as 0.2), *f*_triplets_ is the fraction of triplet exciton in the initial exciton population (0.75), *f*_excitons_ is the formation of excitons from the externally injected charges, and EQE_EL_ is the external quantum efficiency. For the 1-(2,3-dihydrobenzo[*b*][1,4]dioxin-6-yl)-2-(pyren-10-yl)-1*H*-phenanthro[9,10-*d*]imidazole (3)-based device III, the calculated *η*_TTA_ value using the observed data was 54%. The following formula was employed to obtain the quantum yield of TTA (*Φ*_TTA_): *Φ*_TTA_ = *f*_delayed_ EQE/*f*_excitons_*f*_triplets_*f*_outcoupling_. For device III, *Φ*_TTA_ was found to be 15.9%. The luminance–current density curve revealed that TTA was the key factor for the device efficiencies based on pyrenyl-fused polycyclic aryl dihydrobenzodioxin phenanthroimidazole (Fig. 7). Devices I and II exhibited stable external quantum efficiency (*η*_ex_) curves with insignificant roll-off efficiencies, resulting from the balanced carriers mobilities in these devices; however, device III based on 1-(2,3-dihydrobenzo[*b*][1,4]dioxin-6-yl)-2-(pyren-10-yl)-1*H*-phenanthro[9,10-*d*]imidazole showed a roll-off efficiency at low current density (<9 mA cm^−2^), which coincided with Ma *et al.*'s^49^ results about the TTA process. Thiophene-substituted phenanthrimidazole, namely, 1-(4-*tert*-butylphenyl)-2-(5-(pyren-1-yl)thiophen-2-yl)-1*H*-phenanthro[9,10-*d*]imidazole showed a brightness (*L*) of 15 960 cd m^−2^ (4.4 V), current efficiency (*η*_c_) and power efficiency (*η*_p_) of 2.93 cd A^−1^ and 3.02 lm W^−1^, respectively, and external quantum efficiency (*η*_ex_) of 0.88% (Table 2).^72^ The fused polycyclic aryl dihydrobenzodioxin phenanthroimidazole (1–3)-based devices showed excellent efficiencies compared to devices based on thiophene phenanthrimidazoles. The devices with dihydrobenzodioxin phenanthroimidazoles (1–3) showed higher efficiencies than Wang *et al.*’s^73^ device with a configuration of ITO/NPB (40 nm)/Alq_3_ (60 nm)/LiF (1 nm)/Al, which was employed as the reference one (VI).^74^Table 2 shows that the dihydrobenzodioxin phenanthroimidazole-based devices were not inferior to previously reported OLEDs.^75–87^ The outstanding efficiencies indicated that the fused polycyclic aryl phenanthroimidazoles with side-capped dihydrobenzodioxin may be alternative potential emissive materials for OLEDs. Thus further study should be aimed at tuning the fused aromatics at the imidazole carbon of fused polycyclic aryl dihydrobenzodioxin phenanthroimidazoles to further enhance the device efficiencies.

**Table:** Summary of device performances (I–III) and blue OLEDs reported recently

Emitter	*η*_c_ (cd A^−1^)	*η*_p_ (lm W^−1^)	CIE	Ref.
Py-PPI	2.93	3.02	(0.32, 0.59)	72
Py-BPI	3.93	3.20	(0.15, 0.20)	75
TPA-BPI	2.63	2.53	(0.15, 0.09)	63
BPyC	2.94	2.72	(0.15, 0.18)	76
DPEC	4.75	2.55	(0.15, 0.18)	77
Ph3TPE	3.70	2.50	(0.17, 0.20)	78
9TPAFSPO	1.50	2.19	(0.16, 0.07)	79
BPTF	2.06	—	(0.15, 0.13)	80
TPA-AN	4.54	4.02	(0.15, 0.19)	81
MADN	1.43	0.70	(0.15, 0.10)	82
TBADN	2.00	—	(0.16, 0.14)	83
BDMA	2.20	—	(0.16, 0.12)	84
P2	3.08	1.17	(0.17, 0.19)	85
DPF	6.00	3.60	(0.15, 0.19)	86
TPIP	4.69	2.71	(0.15, 0.09)	87
1	4.11	3.80	(0.15, 0.07)	74
2	4.32	3.98	(0.16, 0.14)	74
3	5.20	4.32	(0.16, 0.12)	74
4	6.01	5.01	(0.15, 0.10)	74
5	6.15	5.12	(0.16, 0.08)	74
1	5.10	4.82	(0.16, 0.13)	This work
2	5.35	4.95	(0.15, 0.12)	This work
3	6.90	5.86	(0.15, 0.14)	This work

## 4. Conclusion

Three blue-emissive fused polycyclic aryl phenanthroimidazoles with a side-capping dihydrobenzodioxin moiety showed high thermal device stabilities with efficient device performances. These materials exhibited high *T*_m_ and *T*_d_, as required for device fabrication due to their rigid molecular backbones and non-coplanar arrangement. The devices based on naphthyl and methoxynaphthyl phenanthroimidazoles exhibited stable external quantum efficiency curves with insignificant efficiency roll-offs due to efficient carrier mobilities. Photophysical analysis showed that pyrenyl dihydrobenzodioxin phenanthroimidazole could harvest triplet excitons efficiently through TTA in the EL process. The device with 1-(2,3-dihydrobenzo[*b*][1,4]dioxin-6-yl)-2-(pyren-10-yl)-1*H*-phenanthro[9,10-*d*]imidazole as the buffer layer exhibited a current efficiency of 6.90 cd A^−1^, power efficiency of 5.86 lm W^−1^, external quantum efficiency of 5.30%, and maximum luminance of 53 890 cd m^−2^. Our device efficiencies suggest that pyrenyl dihydrobenzodioxin phenanthroimidazole is an outstanding potential candidate for fabricating efficient blue OLEDs.

## Conflicts of interest

There are no conflicts of interest.

## Supplementary Material

RA-008-C8RA05004J-s001
