# Supplementary material for: Tailoring the molecular design of twisted dihydrobenzodioxin phenanthroimidazole derivatives for non-doped blue organic light-emitting devices
Source: RSC Adv. 2018 Aug 14;8(51):29031–43. doi: 10.1039/c8ra05004j (PMC9084391; doi:10.1039/c8ra05004j)

Electronic Supplementary Material (ESI) for RSC Advances.
This journal is © The Royal Society of Chemistry 2018

Supporting information

Tailoring molecular design of twisted dihydrobenzodioxin phenanthroimidazole derivatives for non-doped blue organic light emitting devices

Jayaraman Jayabharathi*, Ramaiyan Ramya, Venugopal Thanikachalam, Pavadai Nethaji

Department of Chemistry, Annamalai University, Annamalainagar 608 002, Tamilnadu, India

* Address for correspondence

Dr. J. Jayabharathi
Professor of Chemistry
Department of Chemistry
Annamalai University
Annamalai nagar 608 002
Tamilnadu, India.
Tel: +91 9443940735
E-mail: jtchalam2005@yahoo.co.in

Contents

1. **Scheme S1**

2. **Figures S1-S9**

Scheme S1. Synthetic route for fused polycyclic aryl dihydrobenzodioxin phenanthrimidazoles.

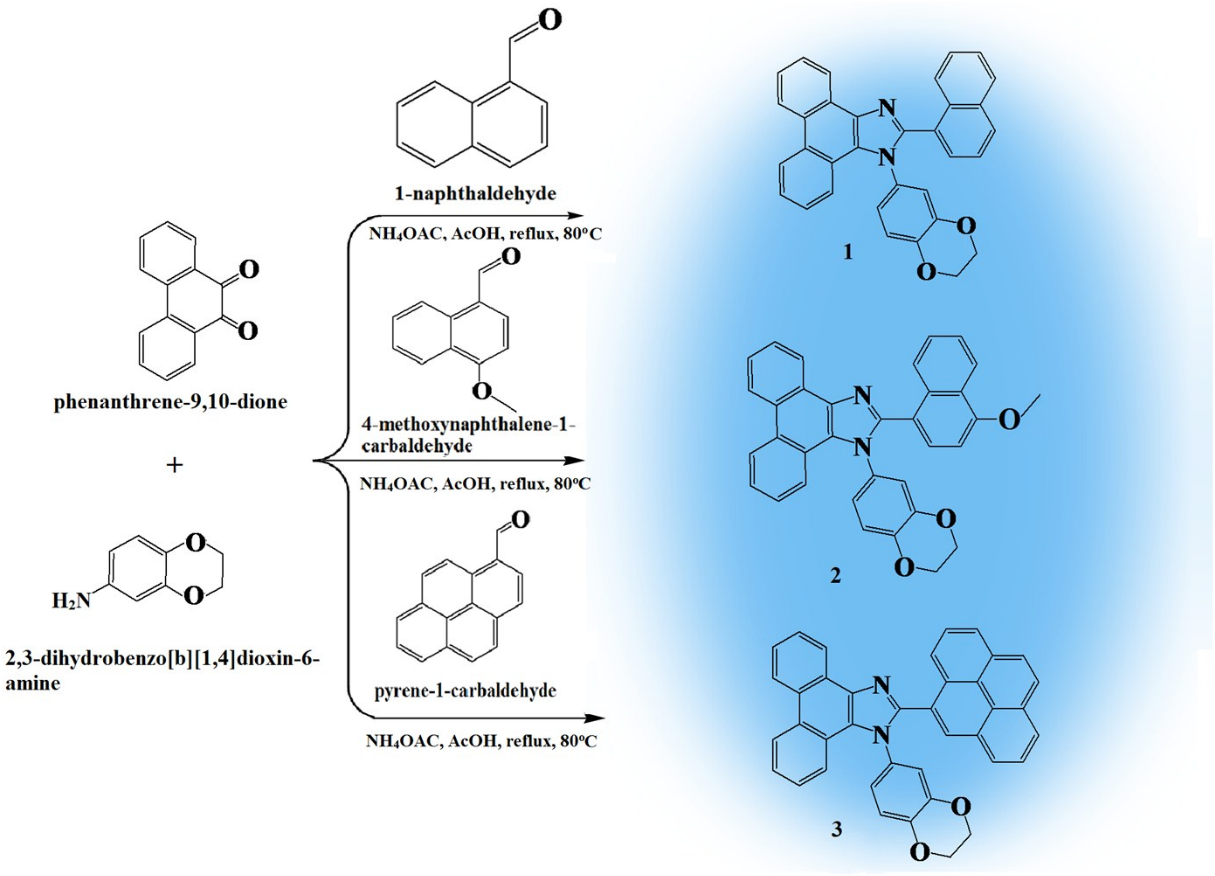

Figure S1: H[1] NMR spectrum of naphthyl dihydrobenzodioxin phenanthrimidazole (1)

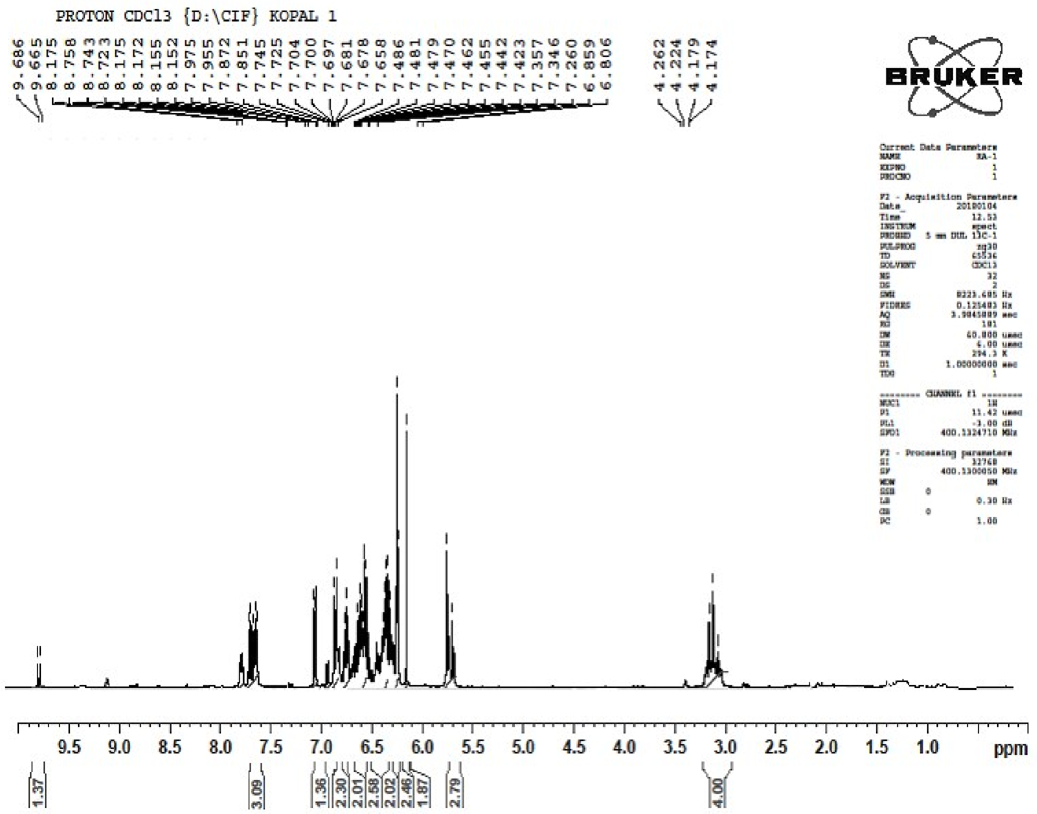

Figure S2: H[1] NMR spectra of methoxynaphthyl dihydrobenzodioxin phenanthrimidazole (2)

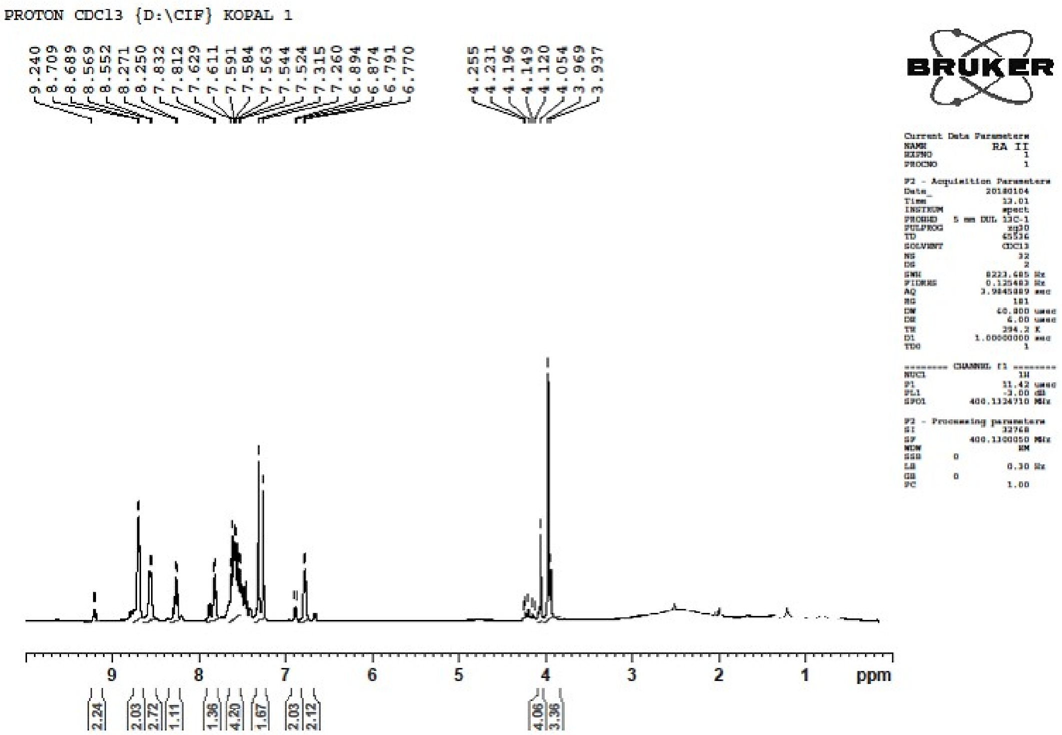

Figure S3: H[1] NMR spectrum of pyrenyl dihydrobenzodioxin phenanthrimidazole (3)

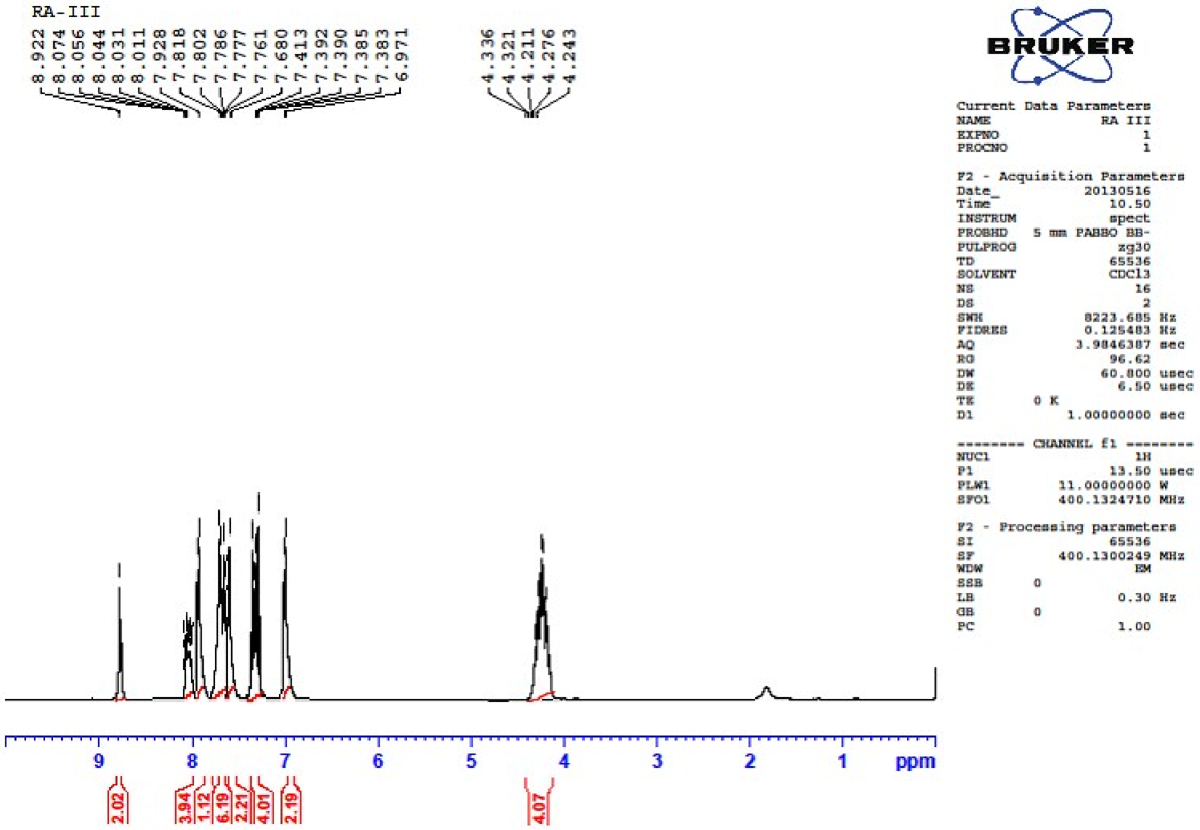

Figure S4: C[13] NMR spectrum of naphthyl dihydrobenzodioxin phenanthrimidazole (1)

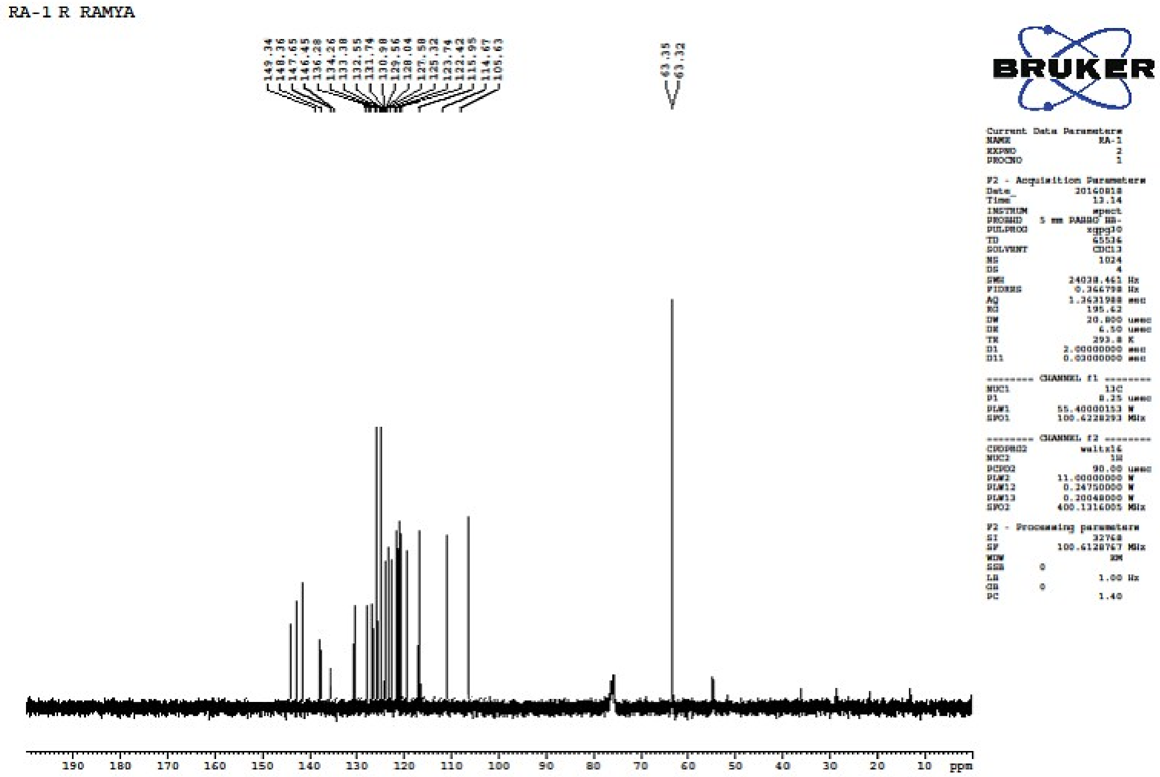

Figure S5: C[13] NMR spectrum of methoxynaphthyl dihydrobenzodioxin phenanthrimidazole (2)

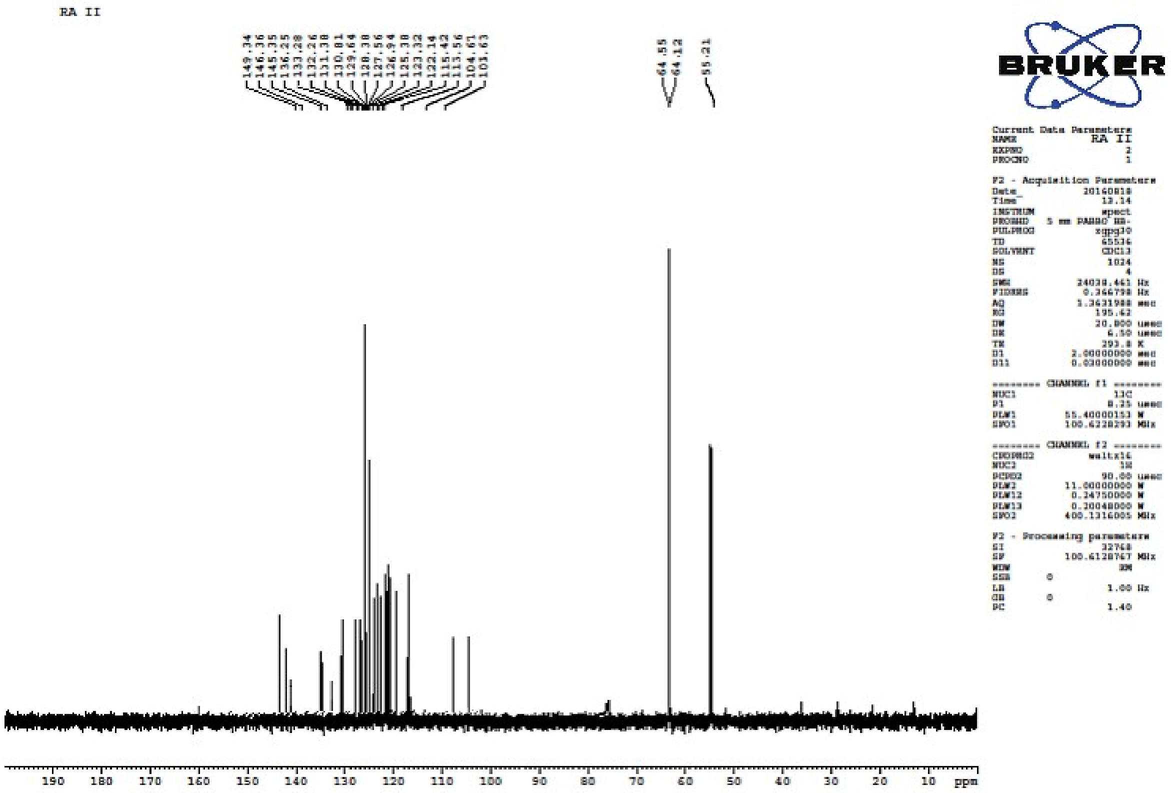

Figure S6: C[13] NMR spectrum of pyrenyl dihydrobenzodioxin phenanthrimidazole (3)

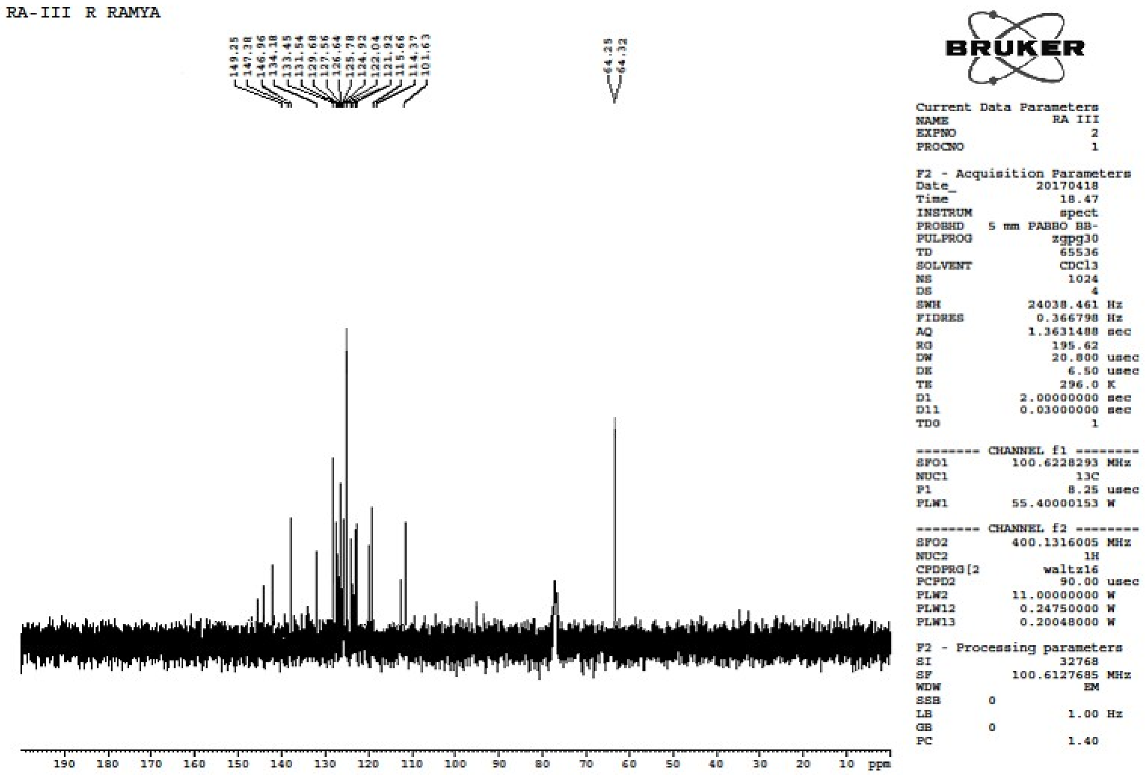

Figure S7: Mass spectrum of naphthyl dihydrobenzodioxin phenanthrimidazole (1)

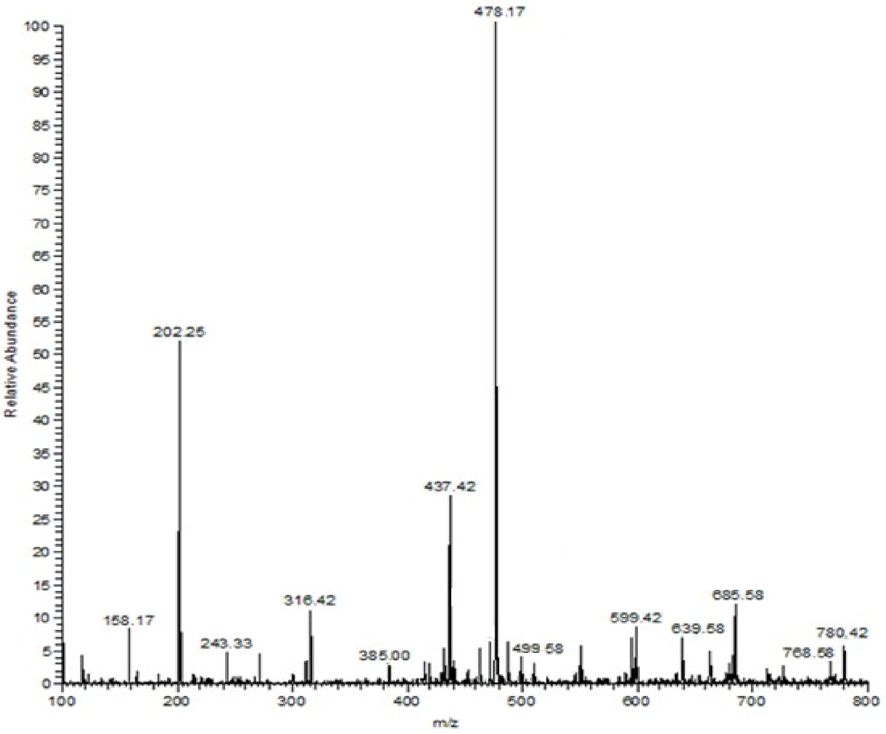

Figure S8: Mass spectrum of methoxynaphthyl dihydrobenzodioxin phenanthrimidazole (2)

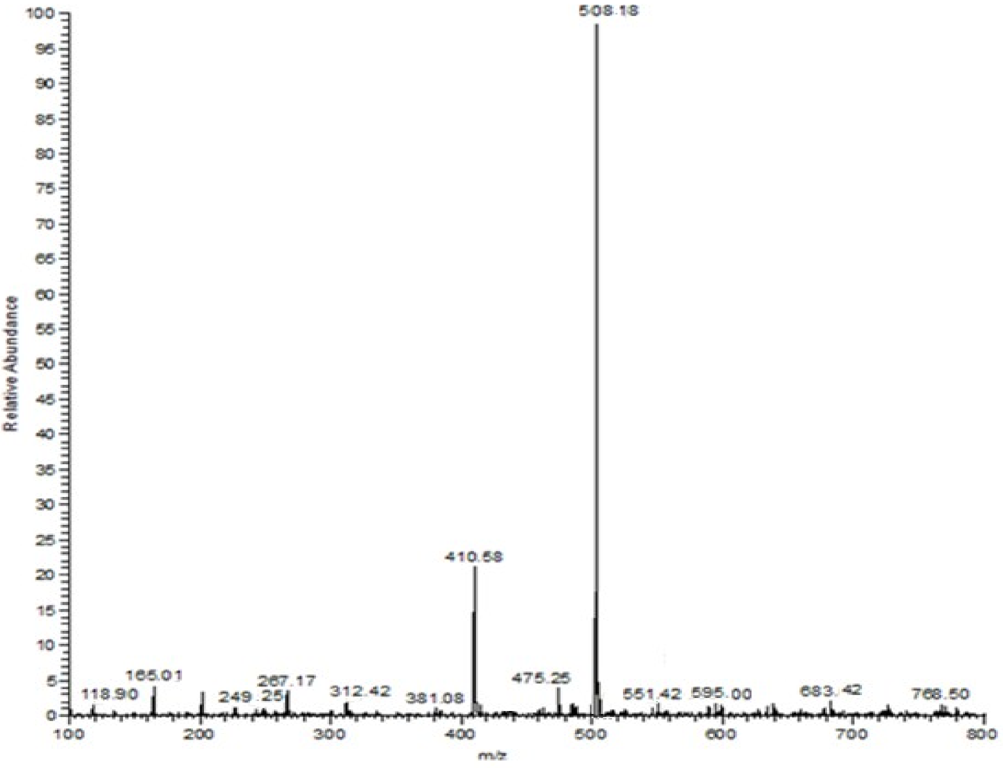

Figure S9: Mass spectrum of pyrenyl dihydrobenzodioxin phenanthrimidazole (3)

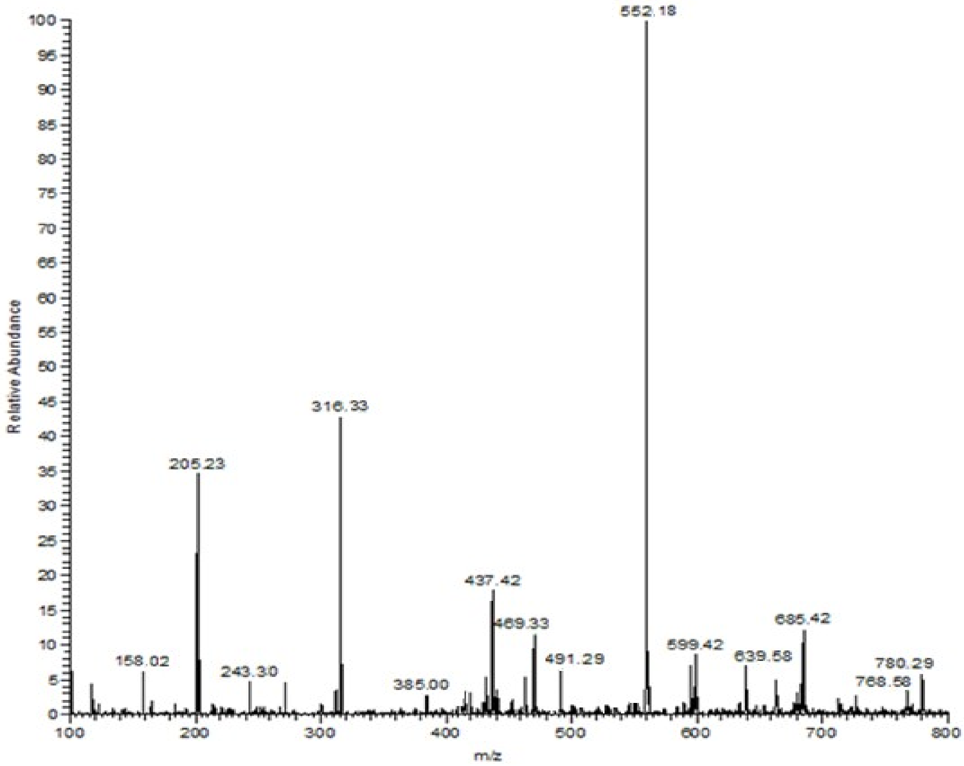

Supplement: RA-008-C8RA05004J-s001 [file RA-008-C8RA05004J-s001.pdf]
